# Anti-allodynic Effect of Mangiferin in Rats With Chronic Post-ischemia Pain: A Model of Complex Regional Pain Syndrome Type I

**DOI:** 10.3389/fphar.2018.01119

**Published:** 2018-10-02

**Authors:** Bárbara B. Garrido-Suárez, Gabino Garrido, Marian Castro-Labrada, Zenia Pardo-Ruíz, Addis Bellma Menéndez, Evelyn Spencer, Jozi Godoy-Figueiredo, Sergio H. Ferreira, René Delgado-Hernández

**Affiliations:** ^1^Laboratorio de Farmacología y Toxicología, Centro de Investigación y Desarrollo de Medicamentos, Havana, Cuba; ^2^Departamento de Ciencias Farmacéuticas, Facultad de Ciencias, Universidad Católica del Norte, Antofagasta, Chile; ^3^Department of Pharmacology, Faculty of Medicine of Ribeirão Preto, University of São Paulo, São Paulo, Brazil; ^4^Centro de Estudio para las Investigaciones y Evaluaciones Biológicas, Instituto de Farmacia y Alimentos, Universidad de La Habana, Havana, Cuba

**Keywords:** adrenergic receptor, chronic post-ischemia pain, complex regional pain syndrome, mangiferin, sympathetically maintained pain

## Abstract

The present study reproduces chronic post-ischemia pain (CPIP), a model of complex regional pain syndrome type I (CRPS-I), in rats to examine the possible transient and long-term anti-allodynic effect of mangiferin (MG); as well as its potential beneficial interactions with some standard analgesic drugs and sympathetic-mediated vasoconstriction and vasodilator agents during the earlier stage of the pathology. A single dose of MG (50 and 100 mg/kg, p.o.) decreased mechanical allodynia 72 h post-ischemia-reperfusion (I/R). MG 100 mg/kg, i.p. (pre- vs. post-drug) increased von Frey thresholds in a yohimbine and naloxone-sensitive manner. Sub-effective doses of morphine, amitriptyline, prazosin, clonidine and a NO donor, SIN-1, in the presence of MG were found to be significantly anti-allodynic. A long-term anti-allodynic effect at 7 and 13 days post-I/R after repeated oral doses of MG (50 and 100 mg/kg) was also observed. Further, MG decreased spinal and muscle interleukin-1β concentration and restored muscle redox status. These results indicate that MG has a transient and long-term anti-allodynic effect in CPIP rats that appears to be at least partially attributable to the opioid and α_2_ adrenergic receptors. Additionally, its anti-inflammatory and antioxidant mechanisms could also be implicated in this effect. The association of MG with sub-effective doses of these drugs enhances the anti-allodynic effect; however, an isobolographic analysis should be performed to define a functional interaction between them. These findings suggest the possible clinical use of MG in the treatment of CRPS-I in both early sympathetically maintained pain and long-term sympathetically independent pain.

## Introduction

Complex regional pain syndrome (CRPS) is a disabling condition most often presenting after trauma affecting the distal part of an extremity. It is characterized by a continuing pain, which is disproportionate to the inciting event, in addition to sensory, autonomic, motor, and trophic disturbances (Bruehl et al., 2002; Beerthuizen et al., 2012). In the clinical setting, it is broadly accepted that the trauma-related differentiation of CRPS is by the absence (CRPS-I) and presence (CRPS-II) of evident nerve lesions. However, a more comprehensive pathophysiology-based classification regarding peripheral inflammatory and central neuroplasticity phenotypes has been proposed (Birklein and Schlereth, 2015). Particularly, the sympathetic activity would be contributory rather than causative and exacerbate ischemia, inflammation, and consequent pain in early-stage disease (Bonica, 1990; Coderre and Bennett, 2010). After trauma, nociceptors in the affected limb become sensitive to catecholamines. Direct or indirect coupling between the efferent sympathetic systems and the afferent systems may lead to sympathetic-maintained pain (SMP). Noradrenaline (NA) released by the sympathetic postganglionic neuron (SPGN) or adrenaline via the circulation are implicated in SMP. Which by means of G protein-coupled receptors (GPCRs) α- and β- adrenoceptors subtypes via distinct signaling pathways modifies the activity of ion channels and Ca^2+^ influx (Pertovaara, 2013). Hypothetical interactions between sympathetic noradrenergic nerve fibers, peptidergic afferent nerve fibers, blood vessels, and immune cells leading to vasoconstriction, further release of cytokines and sensitization, have been suggested. This, in turn, would amplify nociceptive impulse transmission in the spinal cord in CRPS-I patients (Jäning and Baron, 2003). The exaggerated inflammatory response to tissue injury has been suggested to be involved in the origin and maintenance of CRPS-I, since an imbalance between pro-inflammatory with respect to anti-inflammatory cytokine concentrations, elevated activity of mast cells, neurogenic inflammatory reactions, and markers of oxidative stress were found (Huygen et al., 2004; Alexander et al., 2005, 2007; Üçeyler et al., 2007; Birklein and Schmelz, 2008; Eisenberg et al., 2008). Subsequently, the anti-inflammatory treatment of CRPS-I patients may be beneficial and today is a novel tendency that must be investigated (Fischer et al., 2010). Many of the presumably sympathetic symptoms can be explained through inflammation. It has been suggested that the traditionally used sympathetic blocks should be an exception, rather than a rule for CRPS treatment (Birklein and Schlereth, 2015). In view of the positive results in clinical trials for both the ROS scavengers and corticosteroids, studies combining them are recommended (Zollinger et al., 1999; Pérez et al., 2003; Bianchi et al., 2006).

Chronic post-ischemia pain (CPIP) is a recognized model of CRPS-I, which reproduces peripheral pathophysiology after ischemia/reperfusion (I/R) of the hind paw of rodents (Coderre et al., 2004). After reperfusion, animals develop hyperemia and edema for only a few hours; however, the behaviors of spontaneous pain, long-term mechanical and cold hyperalgesia, which spreads to the uninjured contralateral hind paw are significant (Coderre et al., 2004). CPIP rats show a pharmacological profile similar to CRPS patients, which is refractory to most standard analgesic treatments, as well as nociceptive and vascular hypersensitivity to norepinephrine inhibited by anti-sympathetic treatments (Millecamps and Coderre, 2008; Xanthos and Coderre, 2008; Xanthos et al., 2008). In addition, several antioxidants show a long-term anti-allodynic effect in CPIP rats (Kwak et al., 2011; Schiller et al., 2015; Kim et al., 2017; Yeo et al., 2017). I/R leads to the massive appearance of ROS and pro-inflammatory cytokines, which induce microvascular spasms and capillary dysfunction (Coderre and Bennett, 2010). The underlying sex differences in inflammation and oxidative stress which are regulated by estrogens, as well as its impact I/R-evoked mechanical allodynia in CPIP has been reported (Tang et al., 2017). In particular, the NF-κB pathway which induces the transcription of genes implicated in the expression of proteins involved in inflammation, oxidative stress, synaptic plasticity, and neuron–glial interactions is also involved in the development of mechanical allodynia in a CPIP model (de Mos et al., 2009).

Mangiferin (MG) is a glucosylxanthone broadly distributed in higher plants such as *Mangifera indica* L. (Núñez-Sellés et al., 2002). Previous studies have documented the anti-inflammatory and antioxidant properties of MG (Garrido et al., 2004; Pardo-Andreu et al., 2008; Das et al., 2012) without toxic effects (Prado et al., 2015). Furthermore, we proposed its possible value to be investigated in neuropathic pain and CRPS (Garrido-Suárez et al., 2010), since neuroimmune activation and nitroxidative stress are recognized as new targets for its therapeutic intervention (De Leo et al., 2006; Üçeyler et al., 2007; Eisenberg et al., 2008; Salvemini et al., 2011). Additionally, its capacity to modulate endothelial dysfunction is an attractive attribute to the treatment of both early and late stages of CRPS (Song et al., 2015). Most of the biological activities of this compound are explained, at least in part, by inhibition of NF-κB pathway activation (Leiro et al., 2004). MG also shows the ability to decrease mast cell activity related to its anti-allergic properties, as well as neuroprotective and immunomodulatory effects (García et al., 2002; García-Rivera et al., 2006; Campos-Esparza et al., 2009; Pardo-Andreu et al., 2010). Concerning the analgesic profile of MG studied in acute inflammatory pain models, the participation of the endogenous opioid system and adenosine in its anti-nociceptive activity has been accepted (Dar et al., 2005; Lopes et al., 2013). This effect at the peripheral site involves the activation of δ, κ, and probably μ opioid receptors, as well as the L-arginine-nitric oxide (NO)-cGMP ATP-sensitive K^+^ channel pathway (Izquierdo et al., 2013). Moreover, a transient activity of MG on nociceptive pathways mediated by α_2_ adrenergic receptors in cooperation with the opioid system has also been reported (Garrido-Suárez et al., 2014). On the other hand, MG decreased mechano-hypernociception and allodynia in traumatic models of neuropathic pain, also considered as CRPS type II models (Garrido-Suárez et al., 2014; de los Monteros-Zuñiga et al., 2016). Likewise, some preliminary results in clinical CRPS case series treated with *M. indica* extract formulations show an improvement of average pain scores and sensory abnormalities (Garrido-Suárez et al., 2009). These facts make this molecule an attractive multi-target compound with the potentiality to be introduced in CRPS treatment. Then, the aim of the present study was to evaluate the anti-allodynic effect of MG in the early and late stages of CPIP to clarify some underlying pharmacological mechanisms, as well as its potential beneficial interactions with other drugs with clinical relevance on abnormal pain sensations during the earlier stage of the pathology.

## Materials and Methods

### Drugs and Chemicals

Mangiferin (2-β-D-glucopyranosyl-1,3,6,7-tetrahydroxy-9H-xanthen-9-one) was supplied by the Laboratory of Analytical Chemistry, Center of Pharmaceutical Chemistry (Cuba). It was isolated from the *M. indica* stem bark standardized extract by extraction with methanol yielding a yellow powder with 93.82 ± 3.35% purity as determined by liquid chromatographic methods, UV/VIS spectrophotometry and IR, NMR spectroscopic methods and capillary electrophoresis method (Núñez-Sellés et al., 2002). The remaining percentage has been shown to contain a mixture of an isomer, iso-MG (4-C-b-D-glucopyranosyl-1,3,6,7-tetrahydroxyxanthone), and MG monomethyl ether, homo-MG (2-C-b-D-glucopyranosyl-3-methoxy-1,6,7-trihydroxyxanthone) (Núñez Sellés et al., 2016). MG was suspended in DMSO (5% in saline solution) and carboxymethyl cellulose (CMC) 0.05% for intraperitoneal and oral administration, respectively. The solution DMSO + saline has no effects on nociception (Colucci et al., 2008).

Drugs as naloxone hydrochloride, morphine hydrochloride, prazosin hydrochloride, clonidine hydrochloride, yohimbine hydrochloride, 3-morpholinylsydnoneimine chloride (SIN-1), and amitriptyline were purchased from Sigma Chemical Co. (St. Louis, MO, United States). All these compounds were dissolved in saline 0.9%.

Superoxide dismutase was obtained from Randox Labs (Crumlin, United Kingdom), antibodies against rat interleukin-1β (IL-1β) from R&D Systems, Inc. (Minneapolis, MN, United States), Ellman’s reagent [5,5’-dithiobis(2-nitrobenzoic acid)], bovine serum albumin (BSA), glutathione, and malondialdehyde bis(dimethyl acetal) from Sigma Chemical Co. (St. Louis, MO, United States), LPO-586 kit from Calbiochem (La Jolla, CA, United States), and nitrate reductase from Boehringer Mannheim (Milan, Italy). All other reagents were of analytical grade.

### Experimental Animals

Experimental procedures were carried out in accordance with European regulations on animal protection (Directive 86/609), the Declaration of Helsinki, and the Guide for the Care and Use of Laboratory Animals as adopted and promulgated by the US National Institutes of Health (NIH Publication No. 85–23, revised 1996). All experimental protocols were approved by the Institutional Animal Care and Ethical Committee of the Center for Drugs Research and Development (CIDEM/SP00511). Male Sprague-Dawley (8–10 weeks) rats weighing 200–250 g were obtained from the Center for Experimental Animals Production (CENPALAB, La Habana, Cuba). They were kept in controlled conditions (22 ± 0.5°C, relative humidity 40–60%, a 7 a.m. to 7 p.m. alternate light-dark cycle, food, and water *ad libitum*). The experiments took place during the light period and animals belonging to the various treatment groups (n = 6–7 for each group) were tested in randomized order.

### Animal Model of Complex Regional Pain Syndrome Type I (CRPS-I)

Chronic post-ischemia pain was induced by ischemia and reperfusion injury of the left hind paw (Coderre et al., 2004). Briefly, animals were anesthetized over a 3-h period with a bolus (55 mg/kg, i.p.) and chronic i.p. infusion of sodium pentobarbital for 2 h (27.5 mg/kg/h). After induction of anesthesia, a Nitrile 70 Durometer O-ring (O-rings West, Seattle, WA, United States) with a 5.5 mm internal diameter was placed around the rat’s left ankle joint. After 3 h the O-ring was cut, allowing reperfusion of the hind limb. The sham rats received exactly the same treatment, with the exception that the O-ring was cut so that it only loosely surrounded the ankle and did not occlude blood flow to the hindpaw.

### Study Design

#### Treatment With Single Oral Mangiferin on Mechanical Allodynia in the Early Phase of CPIP Model

The effect of single oral doses of MG on mechanical allodynia was studied 72 h post-I/R using four CPIP groups that received MG (10, 50, and 100 mg/kg, 10 mL/kg) or vehicle (CMC 0.05%) and a sham CPIP also treated with vehicle by oral route (*n* = 7 per group). Then five groups were constituted: CPIP-vehicle, CPIP-MG10, CPIP-MG50, CPIP-MG100, and sham-CPIP. Animals were tested in the ipsilateral hind paw at baseline (prior to I/R injury), before treatment, and at 60, 120, and 180 min after treatment.

#### Pre-treatment With α_1_ and α_2_ Antagonists, α_2_ Agonist, Nitric Oxide Donor, Opioid Agonist and Monoamine Reuptake Inhibitor on the Anti-allodynic Effect of Mangiferin in the Early Phase CPIP Model

There were six experiments to examine the MG anti-allodynic mechanisms or its possible beneficial interactions on 72 h post-I/R with drugs utilized in these conditions. In each experiment, different groups of rats were pre-treated with one drug, respectively: prazosin (1–5 mg/kg) a selective α_1_ adrenoceptor antagonist, yohimbine (1–5 mg/kg) a selective α_2_ adrenoreceptor antagonist, clonidine (0.01–0.1 mg/kg) a α_2_ agonist, SIN-1 (1–10 mg/kg) a NO donor, morphine (0.3–3 mg/kg) a preponderantly μ opioid agonist, and amitriptyline (3–10 mg/kg) a monoamine reuptake inhibitor or vehicle (SS). All drugs were administered intraperitoneally (1 mL/kg). Afterward, a single dose of MG (100 mg/kg, i.p.) or vehicle (DMSO 5%) was injected 15 min later. Following 20 min after MG injection and 35 min of the pre-treatment, mechanical allodynia was tested. Approximately 10 min before that, MG or highest doses of agonist drugs (morphine and clonidine) were injected; a group of animals was pre-treated with antagonist drugs naloxone (1 mg/kg) or yohimbine (0.1 mg/kg), respectively. The agonist and antagonist drugs and the doses were selected according to the previous reports and pilot experiments in our laboratory, since all drug doses do not induce significant abnormalities in the rota-rod test (Dar et al., 2005; Millecamps and Coderre, 2008; Xanthos et al., 2008).

#### Treatment With Repeated Oral Doses of Mangiferin on Mechanical Allodynia in the Latest Phase of CPIP Model

A medication protocol to evaluate some clinical effects of MG that could appear only after its repeated oral administration during 7 days after 72 h post-CPIP was also designed. The animals were divided in six CPIP rat groups (*n* = 6–7 each): CPIP-vehicle group that received CMC (0.05%); experimental CPIP-MG groups that received MG (10, 50, and 100 mg/kg, 10 mL/kg; CPIP-MG10, CPIP-MG50, CPIP-MG100); reference group that received prednisone (5 mg/kg; CPIP-P5); and sham CPIP (sham-CPIP) also treated with vehicle by oral route. The rats were evaluated on days 7 and 13 post-I/R injury during medication and after its discontinuation, respectively.

### Mechanical Allodynia

Mechano-allodynia of the hindpaw was assessed by measuring the hindpaw withdrawal response to von Frey filament stimulation according to a modification of the up/down method (Chaplan et al., 1994). Briefly, rats were placed in Plexiglas cages (21 cm × 26 cm × 27 cm) with a wire grid bottom. Filaments (Stoelting, Wood Dale, IL, United States) were applied to the plantar surface of the hind paw for approximately 5 s in either ascending or descending strength, to determine the filament closest to the threshold of response. Each filament was applied five times; a response to three of the five applications was counted as positive. The minimum stimulus intensity was 0.25 g, and the maximum was 15 g. Based on the response pattern and the force of the final filament, the 50% response threshold (grams) was calculated. The resulting pattern of positive and negative responses was tabulated, and the 50% response threshold was interpolated using the formula:

$$50\%\;g\;threshold\;=\;(10^{\left[\mathrm{xf}+k\delta \right]})/10,000$$

where *x*f = the value (in log units) of the final von Frey hair used; *k* = tabular value 11 for pattern of positive/negative responses; and δ = mean difference (in log units) between stimuli (here, 0.224). CPIP rats that had not developed mechanical allodynia at 48 h post-IR injury (non-responders, 50% threshold >10 g) were excluded from further measurements of mechanical allodynia after treatments.

### Tissue Sampling and Preparation

All rats including the control group were euthanized by cervical dislocation under anesthesia with diethyl ether. Each rat was put in a closed cylindrical jar and overdosed with diethyl ether until loss of the righting reflex. This step took approximately 75 s. Short exposure of rats to diethyl ether reported to have an insignificant effect on cytochrome P450 enzymes, which are known to play an important role in oxidative stress (Plate et al., 2005). Then the muscle samples of the superficial plantar layer (one each from the Flexor Hallucis Brevis, Flexor Digiti Minimi Brevis, and Flexor Digitorium Brevis, each weighing between 25 and 50 mg) and spinal cord samples at L5–L6 (each weighing about 20–25 mg) were obtained, cut into small pieces and homogenized using an Omni tissue homogenizer (Omni International, Gainesville, VA, United States) (de Mos et al., 2009). Tissues were homogenized in ice-cold lyses buffer (0.1 M phosphate, pH 7.4, 1 mM EDTA, 9 mL per gram of tissue using a tube pestle). Samples were centrifuged at 1500 × *g* for 15 min at 4°C. The supernatants were then stored at -80°C until further analysis.

### Muscle and Spinal IL-1β Determination by Enzyme-Linked Immunosorbent Assay (ELISA)

Protein was estimated in all homogenates (Lowry et al., 1951). BSA 0.01–0.2 mg/mL was used as standard. Quantification of IL-1β secretion was measured by an in-house sandwich ELISA based on matched antibodies against rat IL-1β. Briefly, a 96-well ELISA plate that was pre-coated with anti-rat polyclonal IL-1β antibody and was incubated with a 100 μL sample or rat IL-1β standards at each well. Following incubation for 2 h at room temperature, the wells were washed three times using a washing buffer, after which biotinylated anti-rat IL-1β antibody was added and incubated for 2 h. Then, streptavidin-peroxidase was added, incubated for another 30 min and followed by three washes. Then a tetramethylbenzidine solution was added, incubated for 30 min in the dark, and the peroxidase-catalyzed color change was stopped by acidification with 1 N H_2_SO_4_. The absorbance was measured with the microplate reader at 450 nm with reference at 630 nm. Results were expressed as pg of cytokine/mg of protein.

### Determination of Redox Biomarkers in Ipsilateral Muscles

Redox biomarkers were determined by spectrophotometric methods using a Pharmacia 1000 Spectrophotometer (Pharmacia, Uppsala, Sweden). Total protein content was measured by Bradford’s method with BSA as standard (Bradford, 1976). SOD activity was determined by using a RANSOD kit, where xanthine and xanthine oxidase were used to generate superoxide anion radicals (O_2_^-^), which react with 2-(4-iodophenyl)-3-(4-nitrophenyl)-5-phenyltetrazolium chloride (INT) to form a red formazan dye. SOD activity was measured by the inhibition degree of this reaction (Boehringer Mannheim, 1987). After precipitation of thiol proteins, the glutathione (GSH) levels were measured according to the method of Sedlak and Lindsay (1968) with Ellman’s reagent [5,5′-dithiobis(2-nitrobenzoic acid)], and the absorbance was measured at 412 nm. Purified GSH was used to generate standard curves. The concentration of MDA was determined using the LPO-586 kit. In the assay, the production of a stable chromophore after 40 min of incubation at 45°C was measured at 586 nm. For standards, freshly prepared solutions of MDA bis [dimethyl acetal] were employed (Erdelmeier et al., 1998). As a surrogate marker of protein damage, carbonyl protein groups were determined as previously described using 2,4-dinitrophenylhydrazine as chromogen (Levine et al., 2000). In addition, nitrates/nitrites (NO_3_^-^/NO_2_^-^) level, as a surrogate marker of nitric oxide (NO), was determined by converting nitrates to nitrites using nitrate reductase. Then, Griess reagent [1% sulphanilamide, 0.1% *N*-(1-Naphthyl)ethylenediamine dihydrochloride in 0.25% phosphoric acid] was added (Granger et al., 1995). Samples were incubated at room temperature for 10 min, and absorbance was measured at 540 nm.

### Statistical Analyses

Data were analyzed using the statistical program Graph Pad Prism 5 (GraphPad Software, Inc., La Jolla, CA, United States). Baseline mechanical allodynia test results were compared with one-way ANOVA followed by Dunnett *post hoc* test. Post-treatment differences between groups were analyzed using a 2-way repeated-measures ANOVA followed by the Bonferroni *post hoc* test. The results are presented as mean ± SEM. Baseline von Frey thresholds are included in the figures for each drug trial but are not included in these statistical analyses. Redox biomarkers and IL-1β were analyzed using one-way ANOVA followed by Dunnett *post hoc* test. The Spearman’s rank correlation coefficient was used to calculate correlations between numerical spinal IL-1β protein data and the paw-withdrawal threshold data. *P ≤* 0.05 was considered statically significant.

## Results

### Effect of Single Oral Mangiferin on Mechanical Allodynia in the Early Phase of CPIP Model

**FIGURE 1:**
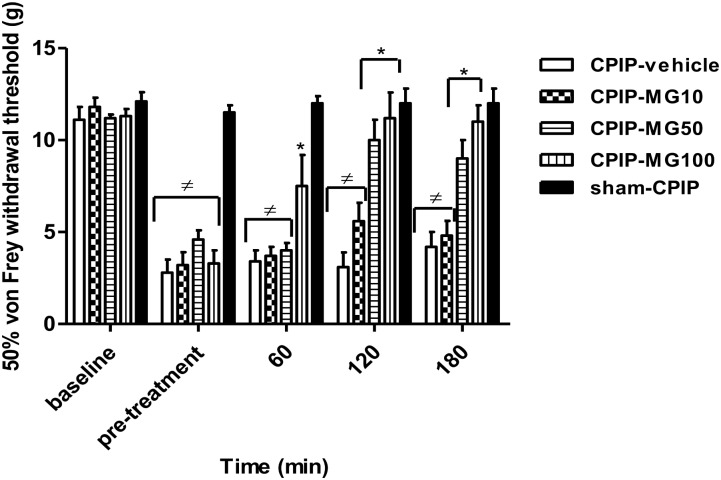
Mechanical ipsilateral paw-withdrawal threshold (grams) determined by von Frey test prior to ischemia/reperfusion injury (baseline), pre-treatment and at 60, 120, and 180 min post-treatment in chronic post-ischemia pain (CPIP) and sham rats. The animals received a single oral dose of mangiferin (10–100 mg/kg, 10 mL/kg, p.o., MG) or vehicle (CMC 0.05%) at 72 h post-reperfusion. Each column represents the reactivity time of 6–7 animals per group as mean ± SEM. ^∗^*P* < 0.05 represents the statistical difference between treated groups and control (CPIP-vehicle), while ^≠^*P* < 0.05 represents the statistical difference between CPIP groups regarding sham-CPIP rats.

Paw-withdrawal thresholds of the ipsilateral hind paw at baseline did not differ between CPIP (*n* = 40) and sham (*n* = 7) rats (11.4 ± 0.2 and 12.1 ± 0.5 g, respectively, *P* = 0.599). At 72 h after I/R injury, CPIP rats developed a reduction in paw-withdrawal threshold (mean 50% von Frey threshold of 4.0 ± 0.3 g) compared to sham (mean 50% von Frey threshold of 11.5 ± 0.3 g) (*P* < 0.001) (**Figure 1**). Within the CPIP group, 33 rats (82.5%) displayed a 50% von Frey threshold <6 and were regarded as responders for mechanical allodynia, which were randomly allocated to different experimental groups. Two-way ANOVA reveals significant main effects of time (pre-post) (*F*_3,93_ = 22.33, *P* < 0.0001) and dose (*F*_4,93_ = 29.28, *P* < 0.0001) and a significant time × dose interaction (*F*_12,93_ = 5.07, *P* < 0.0001). Compared with the vehicle group, the paw-withdrawal threshold only increased significantly at 60 min in CPIP rats treated with a high dose (100 mg/kg) of MG (CPIP-vehicle = 3.4 ± 0.6 g, CPIP-MG10 = 3.7 ± 0.5 g, CPIP-MG50 = 4.0 ± 0.4 g, CPIP-MG100 = 7.5 ± 1.7 g, *P* < 0.05, sham = 12.0 ± 0.4 g). However, at 120 min after its single administration, the anti-allodynic effect was observed in animals that received doses of 50 and 100 mg/kg (CPIP-vehicle = 3.1 ± 0.8 g, CPIP-MG10 = 5.6 ± 1.0 g, CPIP-MG50 = 10.0 ± 1.1 g, *P* < 0.001, CPIP-MG100 = 11.2 ± 1.4 g, *P* < 0.001, sham = 12.0 ± 0.8 g). Likewise, this effect remained at 180 min (CPIP-vehicle = 4.2 ± 0.8 g, CPIP-MG10 = 4.8 ± 0.8 g, CPIP-MG50 = 9.0 ± 1.0 g, *P* < 0.001, CPIP-MG100 = 11.0 ± 0.9 g, *P* < 0.001, sham-CPIP = 12.0 ± 0.8 g) (**Figure 1**).

### Effects of Pre-treatment With α_1_ and α_2_ Antagonists, α_2_ Agonist and Nitric Oxide Donor on the Anti-allodynic Effect of Mangiferin in the Early Phase of CPIP Model

**FIGURE 2:**
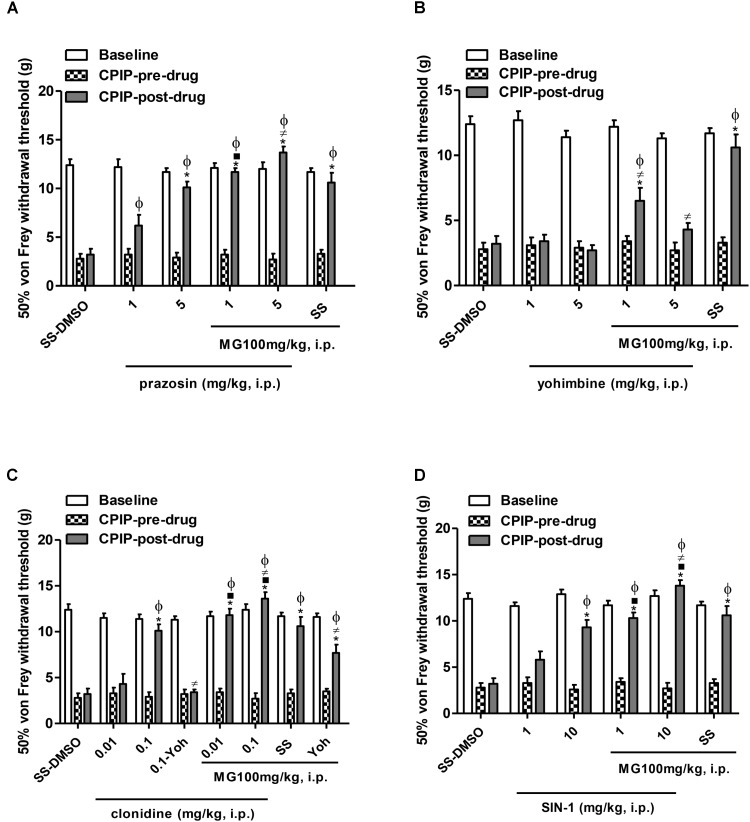
Influence of the pre-treatment with sympathetic α_1_-and α_2_-adrenergic antagonists, prazosin and yohimbine, respectively, α_2_-adrenergic agonist clonidine and nitric oxide donor, SIN-1 (1 mL/kg, i.p.) on the anti-allodynic effect of mangiferin (100 mg/kg, 1 mL/kg, i.p., MG) or vehicle (DMSO 5%) 72 h after the ischemia/reperfusion-injury. Animals received **(A)** saline or prazosin (1–5 mg/kg), **(B)** saline or yohimbine (1–5 mg/kg), **(C)** saline or clonidine (0.01–0.1 mg/kg), **(D)** saline or SIN-1 (1–10 mg/kg). The effect of MG and the highest dose of agonist drug clonidine were reversed by yohimbine (0.1 mg/kg). Each column represents the reactivity time of 6–7 animals per group as mean ± SEM. ^∗^*P* < 0.05 represents the statistical difference between CPIP post-drug and CPIP pre-drug, while ^⋅^*P* < 0.05 represents the statistical difference between CPIP post-drugs in the presence or absence of MG, ^≠^*P* < 0.05 represents the statistical difference between CPIP post-drugs treated with MG or pre-treated with the highest dose of clonidine in presence or absence of yohimbine, ^ϕ^*P* < 0.05 represents the statistical difference between treated groups regarding control vehicle (SS-DMSO).

**Figure 2** shows the mechanical (von Frey) thresholds (grams) of 72 h-CPIP rats at baseline, before, and 35 min after pre-treatment with the vehicle or anti-sympathetic agents (α_1_ and α_2_ antagonists, α_2_ agonist) and a NO donor vasodilator in the presence or absence of MG 100 mg/kg. Two-way ANOVA reveals significant main effects of time (pre-post) (*F*_1,33_ = 415.17, *P* < 0.0001) and treatment (*F*_5,33_ = 14.62, *P* < 0.0001) and a significant time × treatment interaction (*F*_5,33_ = 26.08, *P* < 0.0001). The I/R von Frey thresholds were significantly lower than baselines for all groups and the vehicle (SS – DMSO) injection did not result in a significant increase in paw-withdrawal threshold. MG (100 mg/kg) – SS (pre- vs. post-drug) was found to be significantly anti-allodynic (*P <* 0.001) with respect to vehicle administration (SS – DMSO = 3.2 ± 0.6 g vs. SS – MG100 = 10.7 ± 1.0 g, *P <* 0.001). Compared with pre-drug values, von Frey thresholds were significantly increased in all CPIP groups pre-treated with prazosin, except for its low dose (1 mg/kg) (prazosin 1 – DMSO = 6.4 ± 0.9 g, prazosin 5 – DMSO = 10.1 ± 0.6 g, *P <* 0.001, prazosin 1 – MG = 11.7 ± 0.4 g, *P <* 0.001, prazosin 5 – MG = 13.5 ± 0.3 g, *P <* 0.001). However, a sub-effective dose of prazosin in the presence of MG was found to be significantly anti-allodynic (*P <* 0.001). Additionally, CPIP rats pre-treated with an effective dose of prazosin (5 mg/kg) and treated with MG show significantly increased von Frey thresholds compared to the SS – MG100 group (*P <* 0.05) (**Figure 2A**).

**Figure 2B** shows the (pre- vs. post-drug) comparison of 72 h-CPIP rats mechanical von Frey thresholds of animals pre-treated with yohimbine (1–5 mg/kg). Two-way ANOVA shows significant main effects of time (pre-post) (*F*_1,33_ = 31.45, *P* < 0.0001) and treatment (*F*_5,33_ = 15.61, *P* < 0.0001) and a significant time × treatment interaction (*F*_5,33_ = 10.12, *P* < 0.0001). Non-significant increases in thresholds of animals were observed (yohimbine 1 – DMSO = 3.4 ± 0.5 g, yohimbine 5 – DMSO = 2.7 ± 0.4 g). Nevertheless, in CPIP rats treated with mangiferin, yohimbine reversed its anti-allodynic effect dependently from dose (yohimbine 1 – MG = 6.5 ± 1.0 g, *P <* 0.001, yohimbine 5 – MG = 4.3 ± 0.5 g, *P <* 0.001 vs. SS – MG100 = 10.7 ± 1.0 g).

The results presented in **Figure 2C** show the (pre- vs. post-drug) comparison of 72 h-CPIP rats mechanical von Frey thresholds of animals pre-treated with clonidine (0.01–0.1 mg/kg). Two-way ANOVA shows significant main effects of time (pre-post) (*F*_1,43_ = 244.54, *P* < 0.0001) and treatment (*F*_7,43_ = 25.72, *P* < 0.0001) and a significant time × treatment interaction (*F*_7,43_ = 23.05, *P* < 0.0001). Compared to pre-drug values, von Frey thresholds were significantly increased in all groups pre-treated with clonidine, except the group pre-treated with the low dose (clonidine 0.01 – DMSO = 4.3 ± 1.1 g, clonidine 0.1 – DMSO = 10.1 ± 0.7 g, *P <* 0.001, clonidine 0.01 – MG = 11.8 ± 0.7 g, *P <* 0.001, clonidine 0.1 – MG = 13.5 ± 0.3 g, *P <* 0.001). The particular interest was that a sub-effective dose of clonidine 0.01 mg/kg in the presence of MG was found to be significantly anti-allodynic (*P <* 0.001), this effect was also observed in CPIP rats pre-treated with clonidine 0.1 mg/kg (*P <* 0.01). Additionally, the clonidine 0.1 – MG group shows significantly increased von Frey thresholds compared with SS – MG group (*P <* 0.05). The selective α_2_ antagonist yohimbine 0.1 mg/kg reversed the anti-allodynic effect of the high dose of clonidine (*P <* 0.001) and partially the effect of mangiferin (*P <* 0.05).

The (pre- vs. post-drug) comparison of von Frey thresholds in CPIP rats pre-treated with a donor of NO. Two-way ANOVA shows significant main effects of time (pre-post) (*F*_1,33_ = 252.17, *P* < 0.0001) and treatment (*F*_5,33_ = 16.67, *P* < 0.0001) and a significant time × treatment interaction (*F*_5,33_ = 17.43, *P* < 0.0001). SIN-1 (1–10 mg/kg) show a significant increase in all groups except for its low dose (SIN-1 1 – DMSO = 6.1 ± 0.9 g, SIN-1 10 – DMSO = 9.3 ± 0.8 g, *P <* 0.001, SIN-1 1 – MG = 10.3 ± 0.6 g, *P <* 0.001, SIN-1 10 – MG = 13.8 ± 0.6 g, *P <* 0.001). The sub-effective dose of SIN-1 in the presence of MG was found to be significantly anti-allodynic (*P <* 0.001), this effect was also observed in CPIP rats pre-treated with SIN-1 at a high dose (*P <* 0.01). In addition, the SIN-1 10 – MG group shows significantly increased von Frey thresholds in comparison to the SS – MG group (*P <* 0.05) (**Figure 2D**).

### Effects of Pre-treatment With a μ Opioid Agonist or Monoamine Reuptake Inhibitor on the Anti-allodynic Effect of Mangiferin in the Early Phase of CPIP Model

**FIGURE 3:**
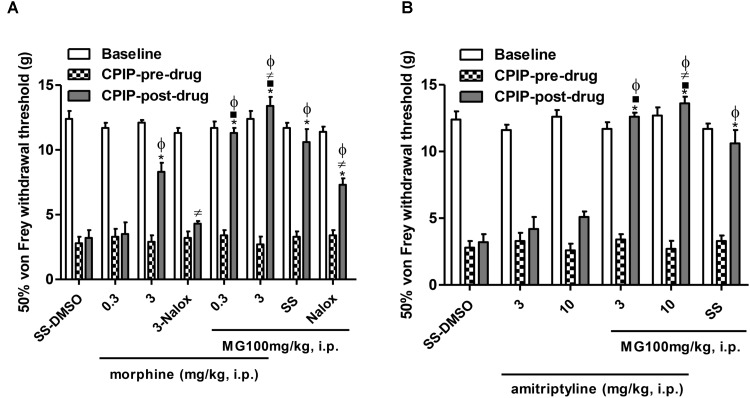
Influence of the pre-treatment with morphine or amitriptyline on the anti-allodynic effect of mangiferin (100 mg/kg, 1 mL/kg, i.p., MG) or vehicle (DMSO 5%) 72 h after the ischemia/reperfusion-injury. Animals received **(A)** saline or morphine (0.3–3 mg/kg), **(B)** saline or amitriptyline (3–10 mg/kg). The effect of MG and the highest dose of agonist drug morphine were reversed by naloxone (1 mg/kg). Each column represents the reactivity time of 6–7 animals per group as mean ± SEM. ^∗^*P* < 0.05 represents the statistical difference between CPIP post-drug and CPIP pre-drug, while ^⋅^*P* < 0.05 represents the statistical difference between CPIP post-drugs in the presence or absence of MG, ^≠^*P* < 0.05 represents the statistical difference between CPIP post-groups treated with MG or highest dose of morphine in the presence or absence of naloxone, ^ϕ^*P* < 0.05 represents the statistical difference between treated groups regarding control vehicle (SS-DMSO).

**Figure 3** shows the mechanical (von Frey) thresholds (grams) of 72 h-CPIP rats at baseline, before, and 35 min after pre-treatment with the vehicle or standard drugs utilized in CRPS patients (morphine or amitriptyline) in the presence or absence of MG 100 mg/kg, 20 min after its injection. Two-way ANOVA shows significant main effects of time (pre-post) (*F*_1,43_ = 235.37, *P* < 0.0001) and treatment (*F*_7,43_ = 19.94, *P* < 0.0001) and a significant time × treatment interaction (*F*_7,43_ = 21.06, *P* < 0.0001). The post-I/R von Frey thresholds were significantly lower than baseline for all groups, and the vehicle (SS – DMSO) injection did not result in a significant increase in paw-withdrawal threshold. SS – MG (100 mg/kg) (pre- vs. post-drug) was found to be significantly anti-allodynic (*P <* 0.001) with respect to vehicle post-drug administration (SS – DMSO = 3.2 ± 0.6 g vs. SS – MG100 = 10.7 ± 1.0 g, *P <* 0.001). The (pre- vs. post-drug) comparison of von Frey thresholds in CPIP rats pre-treated with morphine (0.3–3 mg/kg) show a significant increase in all groups except for its low dose (morphine 0.3 – DMSO = 6.1 ± 0.9 g, morphine 3 – DMSO = 9.3 ± 0.8 g, *P <* 0.001, morphine 0.3 – MG = 10.3 ± 0.6 g, *P <* 0.001, morphine 3 – MG = 13.8 ± 0.6 g, *P <* 0.001). The sub-effective dose of morphine in the presence of MG was found to be significantly anti-allodynic (*P <* 0.001), this effect was also observed in CPIP rats pre-treated with morphine at a high dose (*P <* 0.001). In addition, the morphine 3 – MG group shows significantly increased von Frey thresholds compared to the SS – MG100 group (*P <* 0.05). The non-selective μ opioid antagonist naloxone 1 mg/kg reversed the anti-allodynic effect of the high dose of morphine (*P <* 0.01) and partially the effect of mangiferin (*P <* 0.01) (**Figure 3A**).

Two-way ANOVA shows significant main effects of time (pre-post) (*F*_1,33_ = 204.48, *P* < 0.0001) and treatment (*F*_5,33_ = 31.17, *P* < 0.0001) and a significant time × treatment interaction (*F*_5,33_ = 26.01, *P* < 0.0001). The (pre- vs. post-drug) comparison of von Frey thresholds in CPIP rats pre-treated with amitriptyline (3–10 mg/kg) show a non-significant increase in thresholds of animals (amitriptyline 3 – DMSO = 4.2 ± 0.9 g, amitriptyline 10 – DMSO = 5.2 ± 0.4 g). Nevertheless, both doses of amitriptyline in the presence of MG where found to be significantly anti-allodynic (amitriptyline 3 – MG = 12.6 ± 0.3 g, *P <* 0.001, amitriptyline 10 – MG = 13.6 ± 0.5 g, *P <* 0.001). Additionally, the amitriptyline 10 – MG group shows significantly increased von Frey thresholds compared to the SS – MG100 – group (*P <* 0.05) (**Figure 3B**).

### Effect of Repeated Oral Doses of Mangiferin on Redox Status and IL-1β Concentration in Ipsilateral Plantar Muscle in the Latest Phase of CPIP Model

**FIGURE 4:**
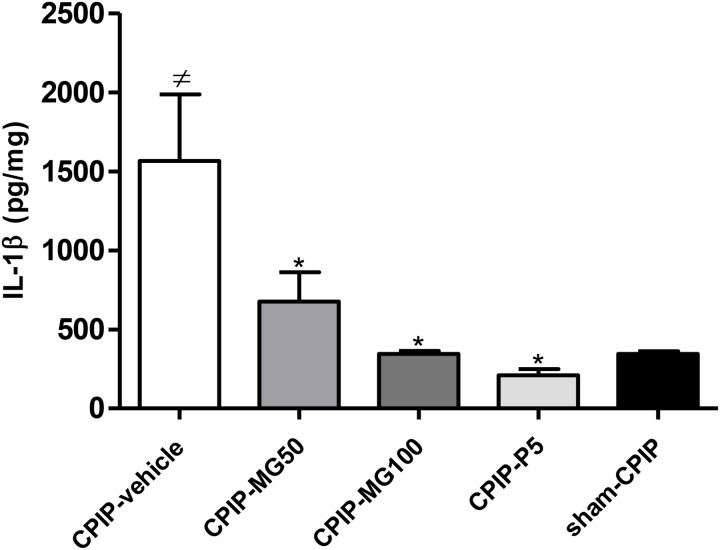
IL-1β protein concentration in the muscle samples of the superficial plantar layer 13 days after post-ischemia/reperfusion and 4 days after mangiferin (10–100 mg/kg, 10 mL/kg, p.o., MG), vehicle (CMC 0.05%) or prednisone (5 mg/kg, P) discontinuation. IL-1β was measured by ELISA. Each bar represents the mean ± SEM (*n* = 6 animals). ^≠^*P* < 0.05 represents the statistical difference regarding sham-CPIP rats, ^∗^*P* < 0.05 represents the statistical difference regarding the CPIP-vehicle group.

Lipid peroxidation (as the concentration of MDA μM/mg Pr in the sample) measured 13 days post-I/R injury was increased significantly (*P* < 0.001) in CPIP rats treated with the vehicle, as compared to sham CPIP controls (**Table 1**). Likewise, a surrogate marker of protein damage (carbonyl protein groups) was increased (*P* < 0.001) in CPIP rats with respect to sham controls. The animals treated with MG 100 mg/kg and prednisone show a significant decrease of MDA formation (*P* < 0.001) and (*P* < 0.01), respectively, as well as CG (*P* < 0.001, *P* < 0.001). A significant increase of NO_3_^-^/NO_2_^-^ levels, as an indicator of NO production in muscle tissue of CPIP rats, was observed (*P* < 0.001). Repeated doses of MG 100 mg/kg like prednisone reduces the NO oxidation products concentrations (*P* < 0.001, *P* < 0.001). GSH was inhibited significantly (*P* < 0.001) in CPIP rats when compared to sham controls. The amount of SOD increased significantly (*P* < 0.001) post-I/R in comparison with the sham CPIP ipsilateral muscle superficial layer. MG 100 mg/kg, like prednisone, significantly restores GSH (*P* < 0.001, *P* < 0.01) and SOD (*P* < 0.001, *P* < 0.001) close to more physiological concentrations. Additionally, IL-1β (pg/mg) in this injured peripheral tissue was also increased in CPIP rats compared with sham controls (CPIP – vehicle = 1565 ± 422 vs. sham – CPIP = 344 ± 17, *P* < 0.001). The experimental groups CPIP – MG100 and CPIP – MG50, as well as referential group prednisone 5, show a significant decrease of this pro-inflammatory cytokine (CPIP – MG50 = 676 ± 185, *P* < 0.05, CPIP – MG100 = 344 ± 21, *P* < 0.001, CPIP – prednisone 5 = 209 ± 39, *P* < 0.001) in ipsilateral muscle tissue 13 days after I/R injury (**Figure 4**).

**Table 1:** Redox biomarkers in ipsilateral muscles of sham and CPIP rats treated with repeated oral doses of mangiferin (MG), prednisone (P), or vehicle.

Group	Dose	MDA (μM/mg Pr)	CG (nM/mg Pr)	NO (μM/mg Pr)	GSH (μM/mg Pr)	SOD (U/L/mg Pr)
Sham-CPIP	10 mL/kg	2.10 ± 0.15	0.71 ± 0.02	14.26 ± 0.74	108.20 ± 5.36	26.33 ± 0.85
CPIP-vehicle	10 mL/kg	5.53 ± 0.32^c^	1.96 ± 0.05^c^	35.26 ± 1.38^c^	61.18 ± 2.38^c^	69.72 ± 1.55^c^
CPIP-P5	5 mg/kg	3.43 ± 0.30^b^	1.19 ± 0.04^a^	17.61 ± 0.51^a^	84.52 ± 3.03^b^	40.09 ± 2.93^a^
CPIP-MG100	100 mg/kg	3.20 ± 0.11^a^	0.94 ± 0.04^a^	24.37 ± 1.28^a^	81.97 ± 1.91^a^	38.91 ± 2.03^a^


*Data are presented as mean ± SEM of *n* = 5–6 per group. ^*a*^*p* ≤ 0.001, ^*b*^*p* ≤ 0.01 represent the statistical difference between treated groups and control (CPIP – vehicle group), and ^*c*^*p* ≤ 0.001 represent the statistical difference between treated groups and sham – CPIP group. CMC 0.05% was used as the vehicle by the oral route. CPIP, chronic post-ischemia pain; CG, carbonyl protein groups concentration; GSH, reduced glutathione; MDA, malondialdehyde; NO, nitric oxide; Pr, protein; SOD, superoxide dismutase.*

### Effect of Repeated Oral Doses of Mangiferin on Mechanical Allodynia in the Latest Phase of CPIP Model and Its Relation to IL-1β Spinal Concentration

**FIGURE 5:**
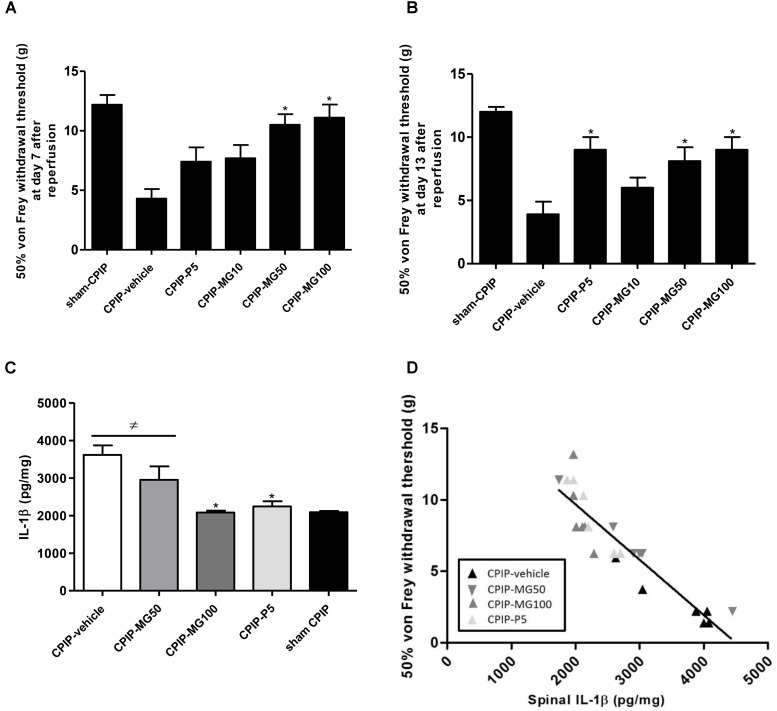
Mechanical paw-withdrawal threshold (grams) determined by von Frey test in CPIP and sham CPIP rats after 7 repeated oral doses of mangiferin (10–100 mg/kg, 10 mL/kg, p.o., MG), vehicle (CMC 0.05%) or prednisone (5 mg/kg, P). **(A)** Effect of MG during the medication at 7 days after ischemia/reperfusion (I/R). **(B)** Effect of MG after its discontinuation 13 days post-I/R. The data are presented as mean ± SEM. ^∗^*P* < 0.05 represents the statistical difference between treated groups and control CPIP-vehicle. **(C)** IL-1β protein concentration measured by ELISA in the spinal cord 13 days post-I/R and 4 days after MG discontinuation. Each bar represents the mean ± SEM (*n* = 6 animals). ^≠^*P* < 0.05 represents the statistical difference regarding sham-CPIP rats, ^∗^*P* < 0.05 represents the statistical difference with respect CPIP-vehicle group. **(D)** The relationship between mechanical paw-withdrawal threshold and the spinal IL-1β concentration 13 days post-I/R injury in CPIP rats that were treated with vehicle, MG 50 mg/kg, MG 100 mg/kg, and prednisone. Rats showed the significant inverse relationship between these variables (*r* = –0.9216; *P* < 0.0001).

At 7 days post-I/R injury and after repeated oral dairy similar doses of MG (10–100 mg/kg) for 5 days, CPIP rats treated with doses of 50 and 100 mg/kg showed an increase in ipsilateral paw-withdrawal threshold compared to vehicle CPIP animals. The group treated with prednisone 5 mg/kg (CPIP-P5) did not significantly increase its von Frey mechanical thresholds at this time (CPIP – vehicle = 4.3 ± 0.8, CPIP – MG10 = 7.7 ± 1.1, CPIP – MG50 = 10.5 ± 0.9, *P* < 0.001, CPIP – MG100 = 11.1 ± 1.1, *P* < 0.001, CPIP – P5 = 7.4 ± 1.2) (**Figure 5A**). Once finished the MG administration at 13 days post-I/R injury, the animals, even after 4 days of its discontinuation, showed significantly increased withdrawal thresholds as well as those treated with prednisone (CPIP – vehicle = 3.9 ± 1.0, CPIP – MG10 = 6.0 ± 0.8, CPIP – MG50 = 8.1 ± 1.1, *P* < 0.05, CPIP – MG100 = 9.0 ± 1.0, *P* < 0.01, CPIP – P5 = 9.0 ± 1.0, *P* < 0.01) (**Figure 5B**). At this time point, IL-1β (pg/mg) in the spinal cord tissue was increased in CPIP rats compared to the sham controls (CPIP – vehicle = 3614 ± 254 vs. sham – CPIP = 2091 ± 33, *P* < 0.01). The concentration of IL-1β in the CPIP-MG100 group (2082 ± 51, *P* < 0.001) decreased significantly, similar to its reference group treated with prednisone (2244 ± 138, *P* < 0.01), compared to the CPIP – vehicle animals, respectively (**Figure 5C**). A Spearman’s correlation analysis for paw-withdrawal threshold data and numerical spinal protein IL-1β data was performed. This analysis included results from the vehicle, prednisone and MG 50 and 100 mg/kg treated CPIP rats (*n* = 24). Correlation analysis identified an association between reduced mechanical paw-withdrawal threshold and increased spinal protein concentration of IL-1β in CPIP rats (*r* = -0.9216; *P* < 0.0001) (**Figure 5D**).

## Discussion

The findings of the present study evidenced for the first time the anti-allodynic effect of MG in CPIP rats. However, the presence of a mixture of an isomer, iso-MG and MG monomethyl ether, homo-MG (Núñez Sellés et al., 2016) could facilitate the pharmacologic effect. This model mimics the CRPS-I-symptomatology made by persistent deep tissue (muscle, bone, and nerve) microvascular pathology that leads to a combination of inflammatory and neuropathic pain processes (Coderre et al., 2004; Coderre and Bennett, 2010). In this context hypoxia and acidosis, as well as the subsequent formation of ROS are robust nociceptive triggers implicated in acute inflammation and edema associated with microvascular endothelial cell I/R injury in the early phase of CPIP (Coderre and Bennett, 2010). These include vascular abnormalities such as persistent ischemia, dependent on either slow-flow no-reflow due to capillary blockage, or arterial vasospasms due to sympathetic vasoconstrictor hyper-responsiveness and/or endothelial cell dysfunction (Blaisdell, 2002). Prolonged deep tissue ischemia for a week following reperfusion may result in chronic inflammation leading to peripheral and central sensitization (Laferrière et al., 2008; Kwak et al., 2011). MG, after its repeated administration, also shows a long-term anti-allodynic effect in the late phase of CPIP related to the decrease of spinal and peripheral IL-β and restoration of redox status in the affected limb.

The sympathetic nervous system (SNS) in CRPS-I may have an important contributory role in early-stage of disease (Coderre and Bennett, 2010). Accordingly, mechanical allodynia in CPIP rats has been explicated in part by sympathetically maintained pain (SMP) mechanisms, particularly exaggerated sympathetically mediated vascular contractility and persistent tissue ischemia (Xanthos and Coderre, 2008; Xanthos et al., 2008). The nociceptive responses to NE in CPIP rats are paralleled by enhanced vasoconstrictive responses to NE and are relieved by α-adrenergic antagonists or vasodilators. Subsequently, an indirect sympathetic–sensory coupling has been proposed to explicate CPIP and CRPS-I hypersensitivity. This process may depend more on early upregulation of adrenoceptors on vascular smooth muscle cells of the rat hind limb than on primary afferents (Xanthos et al., 2008). MG does not affect NE induced vasoconstrictor responses in rat mesenteric resistance arteries, suggesting its inability for antagonized vascular smooth muscle cells α_1_-adrenoceptors, which are pivotal in SMP (Raja et al., 1991; Beltran et al., 2004). However, the inhibition of NE induced vasoconstriction and Ca^2+^ influx in the rat thoracic aorta by its more bioavailable aglycone (norathyriol) and the endothelium-independent vasorelaxant actions of some xanthones have been reported (Ko et al., 1991; Jiang et al., 2004). Several differences between the distribution of sympathetic nerve fibers to the blood vessels have been recognized. For most tissues, all the vessels except the capillaries, precapillary sphincters, and metarterioles are innervated by SNS. Particularly, the smooth muscles in the arterioles of the nutritive capillaries are controlled by local factors, whereas the arterioles in the subpapillary plexus are predominantly sympathetically controlled (Groeneweg et al., 2009). A gradation from proximal to peripheral arteries toward denser innervation and greater neurogenic responses has been found in rats. Then vasoactive effects of individual α-adrenergic receptor subtypes can be strongly influenced by species and expression of subtypes in particular vascular beds. This may be particularly relevant to hindpaw vascular bed under I-R injury which has significant effects within the microvasculature (Nilsson et al., 1986; Xanthos and Coderre, 2008). Despite the fact that the vasoactive effect of MG was studied in large and intermediate arteries it may be an approach to infer its possible activity on vascular α-adrenergic receptors. Sympathetic blocking with systemic guanethidine, α_1_ antagonist prazosin, α_2_ agonist clonidine, and mixed α_1_/α_2_-adrenergic antagonist phentolamine each induce an anti-allodynic effect 2 days after I/R in CPIP rats (Xanthos et al., 2008). Additionally, α_2_ antagonist yohimbine did not induce anti-allodynia, supporting the beneficial role of α_2-_adrenoceptor agonists such as clonidine in CRPS patients (Ragavendran et al., 2016). The present results suggest the contribution of α_2-_adrenoceptors in the anti-allodynic effect of MG as well as any favorable interaction between this xanthone and adrenergic agents. Most α_2-_adrenoceptors are autoreceptors, mediating negative feedback on NE release from SPGNs leading to local vasodilation (Pertovaara, 2013). Nevertheless, spinal α_2_ adrenergic receptors implicated in pre-synaptic and post-synaptic nociceptive inhibition or peripheral α_2_ adrenergic receptors which are expressed by primary nociceptive sensory neurons and immune cells could also be involved in this effect. Previously, a transient effect of MG on nociceptive pathways mediated by spinal α_2_ adrenergic receptors was reported (Garrido-Suárez et al., 2014). Furthermore, the contribution of the L-arginine-NO-cGMP-ATP-sensitive K^+^ channel pathway to its mechanisms (Izquierdo et al., 2013) could have influenced this effect, considering the known reciprocal interplay between NO and α_2_-adrenoceptor in the induction of spinal antinociception (Cury et al., 2011).

The role of the endothelial nitric oxide synthase (eNOS)-NO-cGMP pathway in the regulation of local blood flow and endothelial homeostasis is recognized (Moncada et al., 1991). In the CPIP model, nociceptive behaviors are induced by intradermal injection of the eNOS inhibitor (L-NIO), besides the nociceptive hypersensitivity to NE are reduced by the NO donor (SIN-1) (Xanthos and Coderre, 2008; Xanthos et al., 2008). The current results also suggest a possible beneficial interaction between SIN-1 and MG. After I/R injury, there is both an upregulation and hyper-responsiveness of vascular adrenoceptors as well as a reduced production of NO (Xanthos et al., 2008). In cold CRPS patients, an imbalance between the vasodilator NO and the vasoconstrictor endothelin-1 (ET-1) in artificial blisters on the skin of the affected extremity has been demonstrated (Groeneweg et al., 2006); evidence that advised about the role of endothelial dysfunction linked to aberrant inflammatory responses in CRPS vasomotor disturbances (Groeneweg et al., 2008, 2009). CPIP mice show enhanced painful responses to intraplantar ET-1 injections and upregulation of ET-A receptors in hind paw muscle (Millecamps et al., 2010). Interestingly, a protective effect of xanthones against endothelial dysfunction by reducing the levels of endogenous NOS inhibitors could promote vasodilation in these conditions (Jiang et al., 2004). Congruently, MG dose-dependently enhances eNOS level in the gastric mucosa of an I/R model (Mahmoud-Awny et al., 2015). Likewise, its ability to inhibit smooth muscle spasms in tracheal rings have been linked with epithelium eNOS-NO-cGMP-dependent mechanisms (Vieira et al., 2013). Additonally, it was previously reported that MG inhibited ET-1 secretion and restored the loss of NO production when cells were exposed to high glucose under endoplasmic reticulum stress conditions (Song et al., 2015). An earlier study of the CPIP model revealed that topical combinations including α_2A_ receptor agonists, phosphodiesterase four inhibitors, or NO donors improved both arterial and capillary blood flow associated with effective analgesia (Ragavendran et al., 2013; Laferrière et al., 2014). All these facts add support to our interpretation that MG relaxant properties may be closely related to the activation of the NO-cGMP pathway in CPIP.

Although currently there are no positive trials in CRPS for drugs such as tricyclic antidepressants (TCA) or opioids, both are often indicated (Birklein and Schlereth, 2015). Amitriptyline was ineffective in decreasing mechanical allodynia in CPIP rats and morphine had a slight, but significant anti-allodynic effect, early when tissues exhibit elevated local production of ROS and other inflammatory mediators (Millecamps and Coderre, 2008). This experiment showed that the anti-allodynic effect of MG was naloxone-sensitive. Other authors have described the participation of endogenous opioid peptides and the activation of peripheral opioid receptors in its analgesic mechanisms (Dar et al., 2005; Izquierdo et al., 2013; Lopes et al., 2013). Current results also suggest a beneficial interaction of MG with both drugs, probably related to its ability to modulate noradrenergic, serotonergic, and opioid systems (Garrido-Suárez et al., 2014; de los Monteros-Zuñiga et al., 2016), in addition to a potent antioxidant effect depending on its ROS scavenging (Garrido et al., 2008) and iron-complexing abilities (Pardo-Andreu et al., 2008). Particularly, hypoxia-inducible factor 1α (HIF-1α), which is regulated by ROS, has been implicated in CPIP pathogenesis and its inhibition produces anti-allodynia (Hsiao et al., 2016). MG shows the ability to down-regulate the expression of HIF-1α in ischemic mouse retina (Kim et al., 2016). On the other hand, peroxynitrite (PN; ONOO^-^), may be involved in the mechanical allodynia and NMDA receptor-mediated central sensitization in the CPIP model (Kwak et al., 2014). Subsequently, *N*-acetyl-L-cysteine (NAC) or ONOO^-^ decomposition catalysts prevent mechanical allodynia and decrease the phosphorylation of the NMDA spinal receptor (Kwak et al., 2011, 2014). In agreeing with the present result, a synergistic effect of the combination morphine and NAC, as well as potent analgesia by a bifunctional compound μ opioid agonist/antioxidant, have been demonstrated in CPIP rats (Schiller et al., 2015). In this regard, TCA has a large volume of pre-clinical and clinical evidence, which support the recommendation for its use as a first-line treatment for neuropathic pain (Finnerup et al., 2015). CPIP like CRPS-1 is characterized by the absence of evident nerve lesions (Coderre et al., 2004; Harden et al., 2010). However, the evaluation of anti-neuropathic drugs is rational since a reduced density of the sensory fiber terminal arbors in the epidermis similar to small-fiber neuropathy observed in CRPS-I patients has been described (Oaklander et al., 2006; Laferrière et al., 2008). Additionally, the ectopic discharge of sensory A-fibers and C-fibers traveling within ischemic and inflamed nerves in CPIP play a role in central sensitization (Coderre and Bennett, 2010). Interestingly, MG prevents sciatic nerve Wallerian degeneration, and in clinical settings, the association of *M. indica* extract formulations (containing 10–20% of MG) with amitriptyline improved dynamic mechanical allodynia in CRPS and zoster-associated pain patients (Garrido-Suárez et al., 2009, 2011, 2014).

It has been proposed that the progression from early to late stage CRPS-I reflects the evolving dominance of no-reflow over slow-flow in deep tissue capillaries. The same bases could underlie the transition from SMP to sympathetically independent pain (SIP) in CRPS-I (Coderre and Bennett, 2010). A long-term anti-allodynic effect of MG, even after its discontinuation was also observed, suggesting its utility for preventing the progression from early to late phases of CPIP. Possibly, protective effects against inflammation and vascular abnormalities by repeated treatment with MG from early stages could explicate this result. MG restored the redox balance and decreased IL-1β protein concentration in the ipsilateral paw muscles of CPIP rats. This ability of MG to increase the total antioxidant capacity and GSH, normalize MDA, and reduce a serum level of IL-1β was also previously reported in a gastric ulcer I/R model (Mahmoud-Awny et al., 2015). The rise of pro-inflammatory cytokines has been considered causative of the imbalance between endothelial NO and ET-1 in CRPS, which in turn inhibits eNOS activity and accelerates the transcription of preproendothelin-1 (Patel et al., 2002; Groeneweg et al., 2006). On the other hand, in smooth muscle, cytokines stimulate inducible nitric oxide synthase (iNOS) transcription, which promotes the formation of NO. Subsequently, its combination with superoxide form ONOO^-^ increases oxidative damage and endothelial dysfunction (Alonso and Radomski, 2003). ET-1 and ONOO^-^ also contribute to hyperalgesia in these conditions (Millecamps et al., 2010; Salvemini et al., 2011). In the present study, a reduction of local production of nitrites was observed. MG could restore the physiological balance between endothelial constitutive and inducible NOS in CPIP rats since this xanthone prevented tumor necrosis factor α (TNFα) or IL-1-induced NF-κB activation (Leiro et al., 2004). MG decreases iNOS mRNA and iNOS protein levels in inflammatory peritoneal macrophages, as well as TNFα, NO, and ROS production in several studies (García et al., 2002; Garrido et al., 2004). MG also inhibits IL-1β and iNOS expression in vascular smooth muscle cells (Beltran et al., 2004), besides enhanced eNOS, while reducing iNOS in the serum of rats under gastric I/R insult (Mahmoud-Awny et al., 2015). Inflammatory mechanisms associated with deep tissue nociceptor sensitization and ectopic discharge in sensory fibers could initiate and maintain central sensitization in CPIP (Coderre and Bennett, 2010). Several cytokines including IL-1, IL-6, and TNFα have been found in the spinal cord fluid, serum, and peripheral tissues of CRPS patients (Alexander et al., 2005). TNFα and IL-β have been implicated in mechanical hyperalgesia, as well as inducing long-term potentiation in the spinal dorsal horn via NF-κB in nerve-injured rats (Lin et al., 2007). Pro-inflammatory cytokines and ROS may induce glutamatergic dysfunction for inhibiting glial cells’ ability to remove glutamate from the synaptic cleft (De Leo et al., 2006). Here, 13 days after I/R injury, an inversed correlation between paw-withdrawal threshold and spinal IL-1β protein was observed, while previously the role of muscle and spinal NF-κB in the development of mechanical allodynia in a CPIP model was demonstrated (de Mos et al., 2009). Further non-specific modulators of NF-κB including steroids used as a reference in this study have been useful in animals and CRPS patients (Bianchi et al., 2006). Hence, the anti-allodynic effect of MG could be correlated with the inhibition of spinal IL-1β production.

## Conclusion

The present results suggest that MG displays a transient anti-allodynic effect in CPIP model and any potential favorable interaction with agents that block sympathetic vasoconstriction or enhanced NO-dependent vasodilatation, opioids, and TCA, possibly by additive/or supra-additive mechanisms. However, an isobolographic analysis should be performed to define a functional interaction between them. This effect appears to be at least partially attributable to the opioid and α_2_ adrenergic receptors. Its long-term anti-allodynic effect was related to the spinal and peripheral IL-1β decrease and resorting of redox status in the affected limb, suggesting that MG may be useful in CRPS-I patients with clinical relevance in both SMP and SIP.

## Author Contributions

BG-S provided ideas or concepts for the definition of intellectual context, particularly designed and performed the *in vivo* experiments in CPIP model, analyzed the data, wrote the paper, conceived and designed the project. GG revised the paper, contributed to writing and editing the manuscript, and the execution of project. MC-L performed the analgesic experiments. ZP-R performed the IL-1β determination by ELISA and wrote this method. ABM performed the analgesic experiments. ES performed the analgesic experiments. JG-F performed the redox biomarkers determination in ipsilateral muscles and contributed to execution of project. SF and RD-H supervised the study and contributed to execution of the project. All authors read and approved the final manuscript.

## Conflict of Interest Statement

The authors declare that the research was conducted in the absence of any commercial or financial relationships that could be construed as a potential conflict of interest. The handling Editor and reviewer GY declared their involvement as co-editors in the Research Topic, and confirm the absence of any other collaboration.

## Abbreviations

CPIP: chronic post-ischemia pain
CG: carbonyl protein groups concentration
GHS: reduced glutathione
HPLC: high-performance liquid chromatography
IL-1β: interleukin 1 *beta*
MDA: malondialdehyde
MG: mangiferin
NF-κB: transcription nuclear factor *kappa* B
NMDA: *N*-methyl-D-aspartate
NO: nitric oxide
Pr: protein
ROS: reactive oxygen species
SOD: superoxide dismutase
TNFα: tumor necrosis factor *alpha*
